# Vitamin C Supplementation in Healthy Individuals Leads to Shifts of Bacterial Populations in the Gut—A Pilot Study

**DOI:** 10.3390/antiox10081278

**Published:** 2021-08-12

**Authors:** Antonius T. Otten, Arno R. Bourgonje, Vera Peters, Behrooz Z. Alizadeh, Gerard Dijkstra, Hermie J. M. Harmsen

**Affiliations:** 1 Department of Gastroenterology and Hepatology, University of Groningen, University Medical Center Groningen, 9713 GZ Groningen, The Netherlands; a.t.otten@umcg.nl (A.T.O.); a.r.bourgonje@umcg.nl (A.R.B.); v.peters@umcg.nl (V.P.); gerard.dijkstra@umcg.nl (G.D.); 2 Department of Epidemiology, University of Groningen, University Medical Center Groningen, 9713 GZ Groningen, The Netherlands; b.z.alizadeh@umcg.nl; 3 Department of Medical Microbiology, University of Groningen, University Medical Center Groningen, 9713 GZ Groningen, The Netherlands

**Keywords:** vitamin C, ascorbic acid, gut microbiome, antioxidant, micronutrient, pilot study

## Abstract

Gut microbes are crucial to human health, but microbial composition is often disturbed in a number of human diseases. Accumulating evidence points to nutritional modulation of the gut microbiota as a potentially beneficial therapeutic strategy. Vitamin C (ascorbic acid) may be of particular interest as it has known antioxidant and anti-inflammatory properties. In this study, we investigated whether supplementation with high-dose vitamin C may favourably affect the composition of the gut microbiota. In this pilot study, healthy human participants received 1000 mg vitamin C supplementation daily for two weeks. Gut microbiota composition was analysed before and after intervention by performing faecal 16S rRNA gene sequencing. In total, 14 healthy participants were included. Daily supplementation of high-dose vitamin C led to an increase in the relative abundances of *Lachnospiraceae* (*p* < 0.05), whereas decreases were observed for *Bacteroidetes* (*p* < 0.01), Enterococci (*p* < 0.01) and *Gemmiger formicilis* (*p* < 0.05). In addition, trends for bacterial shifts were observed for *Blautia* (increase) and *Streptococcus thermophilus* (decrease). High-dose vitamin C supplementation for two weeks shows microbiota-modulating effects in healthy individuals, with several beneficial shifts of bacterial populations. This may be relevant as these bacteria have anti-inflammatory properties and strongly associate with gut health.

## 1. Introduction

The gut microbiota consists of a vast collection of bacteria colonizing the gastrointestinal (GI) tract which are pivotal to human health. A disturbed gut microbiota composition occurs in a number of human diseases (often termed “dysbiosis”), characterized by typical shifts of bacterial clusters, e.g., increases in potentially pathogenic bacteria and decreased abundances of commensal, beneficial bacteria [1,2]. Examples of such diseases include inflammatory bowel diseases (IBD), type 2 diabetes mellitus (T2DM), non-alcoholic fatty liver disease (NAFLD) and cardiovascular diseases [3,4]. Recent efforts are increasingly directed towards therapeutic and/or nutritional modulation of gut microbiota as an innovative and promising treatment strategy. Multiple substrates have already been identified as potential health-promoting modulators of the gut microbiota [5]. Interestingly, specific dietary interventions and several types of micronutrients have demonstrated pronounced effects on the gut microbiota, showing both favourable and unfavourable consequences [6,7,8,9,10].

One of the nutritional components that are believed to have potentially beneficial microbiota-modulating effects constitute vitamins. Currently, vitamins are not classified as prebiotics by the International Scientific Association for Probiotics and Prebiotics; however, their ability to alter the gut microbial composition is well-recognised [5,11]. 

Vitamin C (ascorbic acid) is a particularly interesting micronutrient which has known beneficial health effects. Vitamin C is an essential water-soluble vitamin that cannot be endogenously synthesized by humans, but is supplied through diet, mainly via consumption of fruits and vegetables [12,13]. This vitamin is involved in biological processes supporting the immune system, and it has been identified as a main physiological antioxidant in higher-developed organisms [14,15]. Vitamin C supplementation has been reported to exert cell-protective properties against oxidative stress and is thus of particular interest in diseases in which oxidative stress plays a pathophysiological role. However, whether vitamin C has microbiota-modulating properties still needs to be elucidated. 

Here, we designed a pilot intervention study in healthy volunteers to determine whether supplementation with high-dose (1000 mg) vitamin C can favourably alter the composition of the gut microbiota. Vitamin C is proficiently absorbed in the proximal small intestine, as is generally the case with vitamins. A daily intake of 30–180 mg/day leads to 70–90% absorption of vitamin C, yet when applying doses exceeding 1000 mg/day, absorption falls to less than 50% [12,16]. This dose-dependent gastrointestinal uptake in the small intestine allows for delivery of vitamin C to the gut microbiota, which is primarily located in the ileum and colon, when orally administered in high doses of 1000 mg. The aim of this study was to investigate shifts in bacterial populations upon high-dose vitamin C supplementation, which may be a possible beneficial treatment strategy for microbiota-associated diseases.

## 2. Materials and Methods

### 2.1. Study Population

Healthy volunteers aged 18–30 years were included. This pilot study was performed at the University Medical Center Groningen (UMCG), Groningen, the Netherlands, and participants were recruited from March 2017 until May 2017. Subjects were allowed to participate if they fulfilled the following inclusion criteria: age 18–30 years, body mass index (BMI) of ≥18.5 and ≤24.9 kg/m^2^, and self-proclaimed health. Furthermore, it was necessary that participants showed a fine understanding of the study procedures, motivation to comply with study requirements and the willingness to provide written informed consent for participation in the study. Participants were excluded when one (or more) of the following criteria were met: Regular use of dietary supplements (e.g., vitamin C, fish oil) one month prior to screening, known allergies or sensitivities to the study product or any ingredients thereof and use of commercially available probiotic, prebiotic or other supplements that may affect the gut microbiota.

### 2.2. Ethical Considerations

This pilot study was approved by the Institutional Review Board (IRB) (in Dutch: ‘Medisch Ethische Toetsingscommissie’, METc) of the University Medical Center Groningen (UMCG) (IRB ref no. M21.276231 and RR201700217). All subjects provided written informed consent. The study was performed in accordance with the Declaration of Helsinki (2013).

### 2.3. Data Collection and Study Design

At the time of inclusion, standard demographic characteristics, including age, sex, length (cm), weight (kg), body mass index (BMI) (body weight divided by squared height, kg/m^2^), smoking behaviour, alcohol consumption and weekly sports activity were recorded. On days 1 and 3, enrolled participants were instructed to take a baseline faecal sample as control. Participants were instructed to store the samples in their home freezers, immediately after production. Participants were asked to ingest vitamin C 1000 mg daily, for a period of two weeks. Subjects were asked to maintain normal dietary habits during the study. Additional samples were collected on days 15 and 17 after vitamin C supplementation. Frozen faecal samples were transported to the UMCG on dry ice and stored at −80 °C. Compliance to the study procedures was recorded on a predeveloped scheme by the participant. 

### 2.4. Vitamin C Supplement

Patients received daily vitamin C supplementation of the normal diet for a period of 2 weeks. The supplement consisted of 1000 mg of vitamin C (L-ascorbic acid, commercially available) per capsule. 

### 2.5. Sample Processing and Intestinal Microbiota Analysis (16S rRNA Gene Sequencing)

DNA was extracted from faecal samples using the RBB + Qiagen method and quantified by a Nanodrop machine, following the exact same procedure as described previously [17]. Amplification of the genes for the 16S rRNA V3/V4 regions was performed by polymerase chain reaction (PCR), using 2% agar gel electrophoresis (TaKaRa Taq Hot start version kit, Bio Inc., Kusatsu, Japan). The 341F and 806R primers (containing a six-nucleotide barcode and an Illumina-MiSeq adapter sequence) were used (Illumina, San Diego, CA, USA) [17,18,19]. Due to failure of some PCR reactions, DNA samples were cleaned-up using AMPure XP beads (Beckman Coulter, Brea, CA, USA) before repeating the PCR. After successful amplification, concentrations of DNA were measured using Qubit 2.0 Fluorometer (Thermo Fisher Scientific, Waltham, MA, USA). To ensure similar library presentations for each sample, dilutions were prepared accordingly. A library was created by pooling 5 μL of each DNA sample as input for 16S rRNA amplicon sequencing (MiSeq Benchtop Sequencer, Illumina, San Diego, CA, USA). Samples with low DNA concentrations after clean-up (quality score < 0.9) were excluded from further analysis. Taxonomic profiling was performed using PAired-eND Assembler for DNA sequences (PANDAseq) and Quantitative Insights into Microbial Ecology (QIIME) [17,20,21]. This procedure was used to assign bacterial taxonomy up to family and genus level, and ARB was used to further identify sequences to species level [22]. Finally, relevant bacterial groups and bacterial species were extracted from the data for downstream analysis, which were selected based on their relative abundance and their importance with regard to vitamin metabolism and observed alterations in human diseases.

### 2.6. Statistical Analysis

Descriptive data were presented as medians with interquartile ranges (IQR) for continuous variables and proportions *n* with corresponding percentages (%) in case of nominal variables. From the original QIIME raw data, relative abundances of bacteria and groups of bacterial families were extracted. Differences in relative abundances of bacterial groups before and after intervention were compared using Wilcoxon signed-rank tests. Hierarchical agglomerative clustering (HAC) analysis was performed by computing pairwise distances between observations using Euclidean distances and visualizing these using the *clustermap* function of the *seaborn* package in Python. Clustering was performed to identify distinct bacterial clusters and shifts of these clusters before and after vitamin C supplementation. Correlations between bacterial relative abundances were determined using Spearman’s rank correlation coefficients (*ρ*), presented in a correlation matrix and visualized within a heatmap. Two-tailed nominal *p*-values ≤ 0.05 were considered as relevant trends, whereas false discovery rates (FDR) < 0.1 after adjustment for multiple comparisons (Benjamini–Hochberg method) were considered statistically significant. Statistical analyses were performed using the Python programming language (v.3.8.5, Python Software Foundation, https://www.python.org, accessed on 18 June 2021), using the *pandas* (v.1.2.3), *numpy* (v.1.20.0) and *statsmodels* (v.0.12.2) modules. Data visualization was performed using the *seaborn* (v.0.11.1) and *matplotlib* (v.3.4.1) packages in Python.

## 3. Results

In total, 14 participants were included who completed the full study programme and whose samples passed quality control after 16S rRNA gene sequencing data processing. No adverse events occurred in this pilot study.

### 3.1. Baseline Characteristics of the Study Population

Baseline demographic characteristics and lifestyle habits are shown in Table 1. Median age of participants was 22 years (IQR: 21–23), and gender distributions were almost equal (males: 43% vs. females: 57%). Only two participants (14%) were smokers, and median alcohol consumption was 5 units per week. Participants were moderately physically active with a median of 3 (IQR: 2–6) hours of exercise per week.

### 3.2. Changes in Bacterial Groups after Vitamin C Supplementation

**Figure 1:**
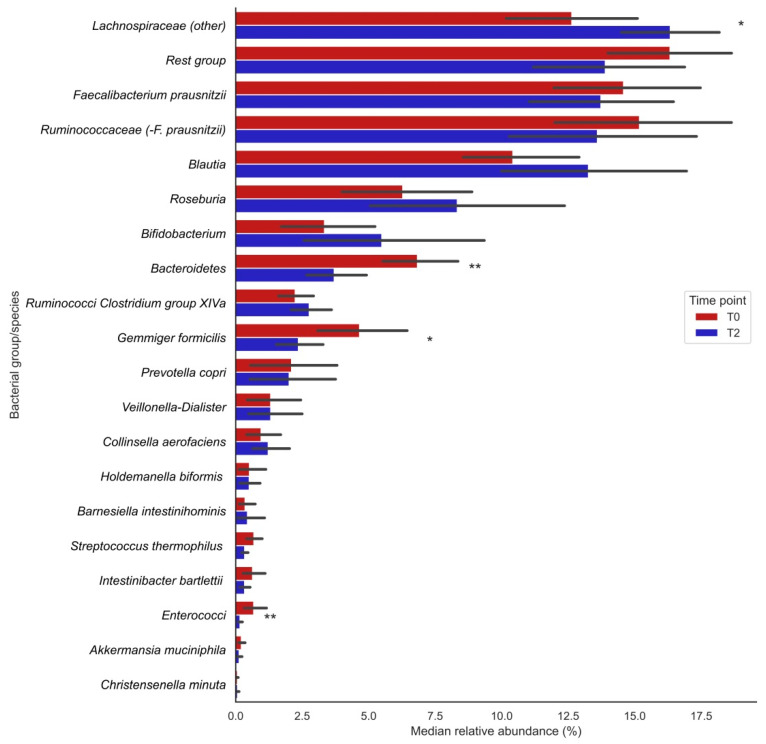
Changes in median relative abundances (%) of bacterial groups before (T0) and after (T2) vitamin C supplementation. Bars indicate median relative abundances of bacterial groups with a corresponding IQR (black lines). * Nominal *p*-value ≤ 0.05; ** Nominal *p*-value ≤ 0.01.

Analysis of 16S rRNA gene sequencing data showed that the relative abundances (%) of the bacterial groups *Lachnospiraceae* (other), *Bacteroidetes*, enterococci and the species *Gemmiger formicilis* were significantly changed after vitamin C supplementation (visualized in Figure 1; the exact numbers and corresponding *P*-values can be found in Table 2). The relative abundances of *Lachnospiraceae* significantly increased after supplementation (T0: 12.7 [9.71;16.6] vs. T2: 16.2 [13.8;17.6], *p* = 0.013), whereas those of *Bacteroidetes* (T0: 5.97 [4.88;8.87] vs. T2: 3.09 [2.14;4.77], *p*= 0.004), *Gemmiger formicilis* (T0: 3.96 [2.51;6.59] vs. T2: 1.86 [0.81;3.68], *p* = 0.013) and enterococci (T0: 0.40 [0.22;0.51] vs. T2: 0.13 [0.07;0.18], *p* = 0.009) significantly decreased after supplementation (Table 2). Furthermore, trends for bacterial shifts were observed for *Blautia* (nominal *p* = 0.035), *Ruminococcaceae Clostridium* group XIVa (*p* = 0.035) and *Streptococcus thermophilus* (*p* = 0.096). No particular trends of bacterial shifts were observed for smoking participants.

**Figure 2:**
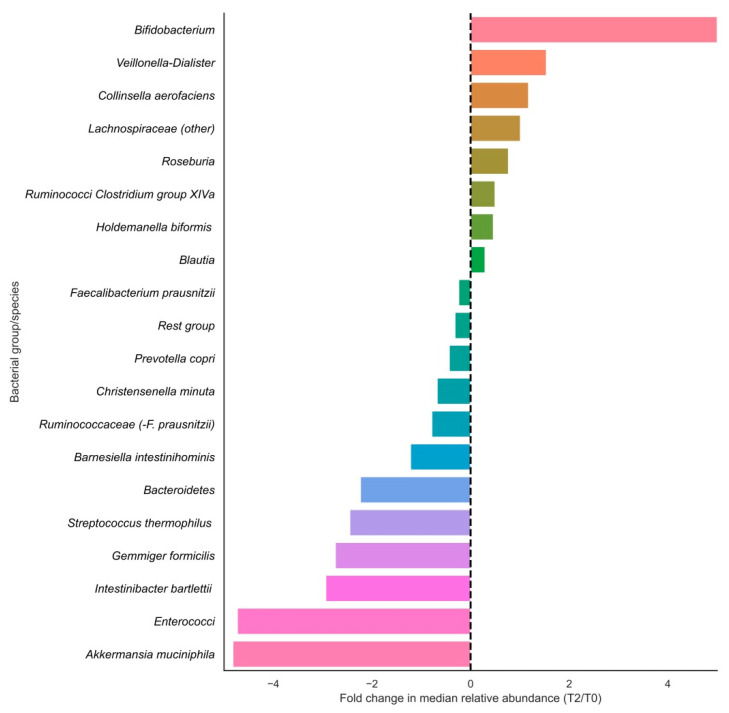
Fold changes in relative abundances (%) of bacterial groups before and after vitamin C supplementation. Bars indicate fold changes of bacterial groups after supplementation (T2) compared to the relative abundance at baseline (T0). Relative increases in bacterial abundance are depicted on the right side of the plot, whereas decreases are shown on the left side of the plot. Bacterial groups are sorted on fold change sizes.

In terms of relative changes in bacterial group abundances, highest fold changes in relative abundances in the positive direction were observed for *Bifidobacteriaceae*, *Veillonella-Dialister*, *Collinsella aerofaciens*, *Lachnospiraceae (other)*, *Roseburia* and Ruminococci from *Clostridium* group XIVa, and largest fold changes were observed in the negative direction for *Akkermansia muciniphila*, enterococci, *Intestinibacter bartlettii*, *Gemmiger formicilis* and *Streptococcus thermophilus* (Figure 2). Of these fold changes, only those of *Lachnospiraceae (other)*, *Gemmiger formicilis, Bacteroidetes* and Enterococci were statistically significant (*p* < 0.05).

### 3.3. Characterization of Bacterial Clusters before and after Supplementation

**Figure 3:**
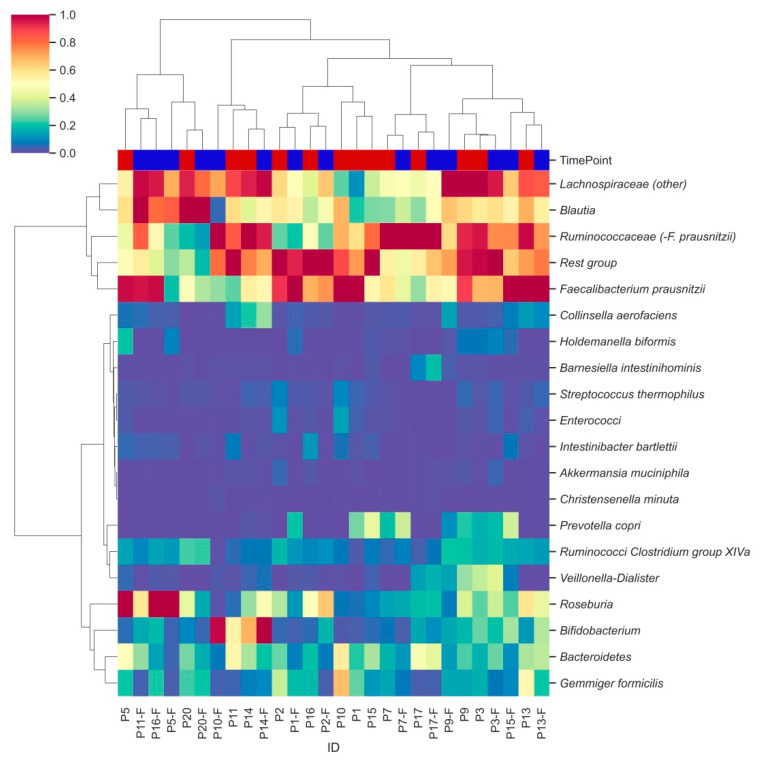
Hierarchically clustered heatmap showing distinct bacterial clusters across participants’ faecal samples. Heatmap columns are coloured by the time point (T0: red; T2: blue) from which each sample was derived. Hierarchical clustering was performed using pairwise Euclidean distances across sample observations. Colouring was based on relative abundances set on a standardized scale.

Hierarchical clustering analysis identified two major clusters of bacterial groups, one of which is characterized by the combination of *Lachnospiraceae*, *Blautia*, *Ruminococcaceae* and *Faecalibacterium prausnitzii* (Figure 3, row level dendrogram). In the second cluster, subclusters were observed for Ruminococci from Clostridium group XIVa and *Veillonella*-*Dialister*, as well as for *Gemmiger formicilis* and *Bacteroidetes*. Remaining bacterial groups were less abundant and did not show distinct clustering. Moderate clustering was observed between samples from the two different time points (column labels, Figure 3).

**Figure 4:**
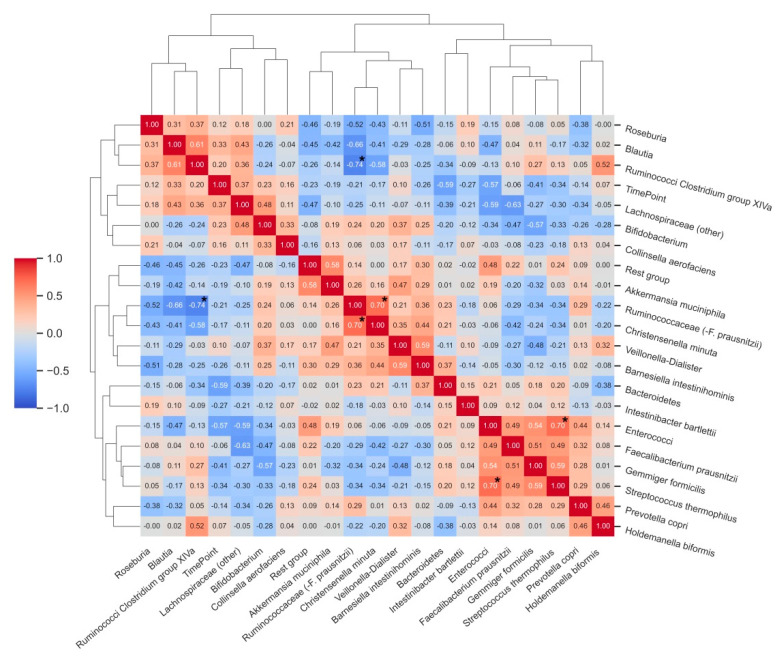
Correlation matrix heatmap demonstrating correlations between relative abundances (%) of bacterial groups before and after vitamin C supplementation. Time point (T0 and T2) was included as an additional variable to the correlative clustering as well as to demonstrate associations between bacterial groups and changes upon intervention. Dendrograms represent hierarchical agglomerative clustering based on Euclidean distances (same as in Figure 3). * Bold asterisks indicate statistically significant correlations after adjustment for multiple comparisons.

Many correlations were observed among the analysed bacterial groups, although few of them reached statistical significance after adjustment for multiple comparisons (Figure 4). Statistically significant correlations were found between the relative abundance of enterococci and *Streptococcus thermophilus (ρ =* 0.70, *p* < 0.05), Ruminococcaceae (−*F. prausnitzii*) and *Christensenella minuta* (*ρ* = 0.70, *p* < 0.05), and there was an inverse correlation between the relative abundance of Ruminococci from Clostridium group XIVa and Ruminococcaceae (−*F. prausnitzii*). Relative abundances of *Bacteroidetes* (*ρ* = −0.59) and enterococci (*ρ*= −0.57) showed strong, inverse associations with the T2 time point of the study (after vitamin C supplementation).

## 4. Discussion

In this human pilot intervention study, high-dose vitamin C supplementation for two weeks led to substantial shifts in faecal bacterial populations. In particular, we demonstrated a significant increase in the median relative abundance of the *Lachnospiraceae* family and trends towards an increased relative abundance of the genus *Blautia.* In contrast, significant decreases in median relative abundances were observed for the phylum *Bacteroidetes* and genera enterococci after vitamin C supplementation. The bacterial species *Gemmiger formicilis* also demonstrated a significant decrease in median relative abundance. Taken together, these results suggest that two-week high-dose vitamin C supplementation may confer beneficial alterations in gut microbial populations, while leaving only a few exceptions.

The results of this study are relevant for a variety of reasons. Currently, human clinical studies investigating the effect of vitamins on the gut microbiota are sparsely available. Previous clinical studies investigating vitamin B_2_ and vitamin D supplementation demonstrated potential beneficial alterations in microbiota compositions [23,24]. More recently, a study in healthy volunteers investigating the effects of colon-delivered vitamins A, B_2_, C, D and E on the gut microbiota reported microbiota-modulating effects of vitamins B_2_, C and D. Vitamin C supplementation (500 mg/day for 4 weeks) demonstrated increased microbiota diversity and an increased abundance of *Collinsella* [25]. The advantage of oral high-dose vitamin C supplementation over controlled colon-delivery of vitamin C lies in the combination of its absorption in the upper gastrointestinal tract while concurrently establishing vitamin C delivery to the gut microbiota, as high-dose vitamin C is only partially absorbed in the small bowel [12]. This approach may be of particular value for patients with diseases that might benefit from microbiota-modulation and who are simultaneously at risk of vitamin C deficiency. 

No studies have followed to investigate whether commercially available high-dose vitamin C supplementation has the capacity to alter the gut microbiota. The strength of our study primarily lies in its novelty. Apart from that, our results may be of interest to a multitude of medical disciplines. For instance, neurodegenerative diseases, such as Parkinson’s disease (PD), are characterized by disturbed gut microbial composition and functionality. A previous study in patients with PD showed that abundances of the bacterial genera *Blautia* and *Roseburia,* two bacterial groups whose presence could potentially be increased through vitamin C supplementation, were markedly decreased compared to healthy individuals [26]. Furthermore, in patients with liver cirrhosis, an increased abundance of the genus *Blautia* was found to be associated with favourable disease outcomes, whereas overrepresentation of the genus *Enterococcus* was linked to inflammation and a reduction in cognitive function [27]. 

At the same time, however, some limitations of this study also warrant recognition. First, this study was of explorative origin as a pilot study, in which only 14 healthy volunteers were included. Therefore, our results should be interpreted with caution as these should be confirmed and validated in larger study populations, consisting of more follow-up data. Second, although the observed changes in bacterial populations may suggest potential beneficial effects to the gut microbial community, they confer limited generalizability due to (1) our strict inclusion and exclusion criteria, (2) the fact that gut microbiota in diseased circumstances may already be in an altered state, and (3) the fact that the gut microbiota may respond differently to nutritional interventions between individuals. Another limitation of our study included the relatively short duration of follow-up, preventing us from studying long-term effects of high-dose vitamin C supplementation on the gut microbiota composition as well as the absence of additional biological health parameters (e.g., inflammatory or oxidative stress biomarkers), which may have sustained the observed potentially beneficial effects. We have not performed measurements related to the gut metabolome, which may provide valuable information regarding microbial functions [28]. However, Pham et al. [25] reported a significant increase in total faecal SCFA concentrations after vitamin C supplementation, which corresponds with the shift towards butyrate-producing bacteria as observed in our study.

The strongest effect of high-dose vitamin C supplementation was the increased relative abundance of the family *Lachnospiraceae*. The *Lachnospiraceae* bacterial family belongs to the phylum Firmicutes and is a predominant bacterial group of the gut microbiota in healthy subjects [29,30]. *Lachnospiraceae* form one of the main producers of short-chain fatty acids (SCFAs). These are bacterial metabolites that are known to possess several anti-inflammatory and antioxidant effects [31]. A study investigating the effect of micronutrients on the gut microbiota composition of patients with cystic fibrosis found that increased vitamin C intake positively correlated with the abundance of Firmicutes [32]. The same study found a negative correlation of vitamin C with abundance of the phylum *Bacteroidetes,* a finding which is also supported by the present study. The exact mechanisms of interaction between the *Lachnospiraceae* family and its human host remain to be determined. As is often the case with specific bacterial groups, it remains largely unclear whether an increased or decreased abundance may be a cause or consequence of the disease. For instance, a greater abundance of *Lachnospiraceae* is found in patients with T2DM and liver and kidney diseases [29]. Interestingly, a decreased abundance of *Lachnospiraceae* is found in faecal samples of Crohn’s disease (CD) and ulcerative colitis (UC) patients [29,33].

In this study, we demonstrated an approximately 4-fold decrease in the median relative abundance of *A. muciniphila.* This is a strictly anaerobic, mucin-degrading bacterial species that has been reported to support immunological and metabolic responses of its host [34] and is of interest due to a potential role as a probiotic [35]. However, decreased abundances of *A. muciniphila* are usually inversely associated with disease states, e.g., T2DM, IBD and obesity [34].

Vitamin C is particularly known for its antioxidant properties, and it belongs to the antioxidant machinery, where it is part of the pool of nonenzymatic antioxidants such as vitamin E (alpha-tocopherol), polyphenols (e.g., *N-acetylcysteine*, NAC) and uric acid [36]. These substances are able to modulate oxidative stress by scavenging reactive oxygen species (ROS). In a variety of disease contexts, vitamin C has been reported to decrease indicators of oxidative stress and inflammation [37,38], but further high-quality clinical trials with relevant endpoints are needed to establish the true role of vitamin C as an antioxidant supplement [39,40]. The direct effect of vitamin C on the intestinal redox balance presumably plays an important gut microbiota-modulating role. Faecal redox potential is dose-dependently related to the ratio of relative abundance of aerotolerant to strict anaerobic species [41]. Strictly anaerobic gut bacteria, such as the bacterial family *Lachnospiraceae* and the species *Faecalibacterium prausnitzii,* which belong to the Healthy Human Mature Anaerobic Gut Microbiota (HMAGM), are profoundly influenced by oxidative stress [41,42]. The presence of oxygen in the intestinal lumen can promote the growth of *Enterobacteriaceae*; whereas it compromises the growth of *F. prausnitzii,* as this bacterial species is particularly susceptible to oxygen [43,44,45].

Vitamin C supplementation could be of particular interest for patients with inflammatory bowel disease (IBD). Associations between IBD and imbalances of gut microbiota composition are becoming increasingly apparent. Homeostasis of the gut microbiota is hypothesized to play a pivotal role in pro-inflammatory and anti-inflammatory responses in the intestines [46,47]. IBD is characterized by chronic inflammation of the gastrointestinal tract. IBD is typically diagnosed in young adulthood and requires lifelong medical therapy [48,49]. The bacterial family *Lachnospiraceae* and the genus *Blautia* (increased relative abundance in our study) have been found to be reduced in abundance in patients with Crohn’s disease [50,51] and the bacterial genus *Enterococcus* and species *G. formicilis* (decreased relative abundance in our study) have been associated with recurrent and relapsing CD [52,53]. A reduction in *Lachnospiraceae* abundance is associated with attenuated butyrogenesis, which is linked to a possible recurrence of UC [33]. However, the literature is inconsistent about the effect of low levels of *Bacteroidetes* found after vitamin C supplementation. Enrichment of *Bacteroidetes* has been shown to be associated with low-grade systemic inflammation, yet increased *Bacteroidetes* abundance is also associated with decreased colitis development during cancer treatment with ipilimumab [54]. Low levels of genus *Bacteroides* have been associated with IBD, and a decreased abundance was more noticeable during active disease when compared to quiescent disease [55]. Contrarily, studies have associated an increased abundance of *Bacteroides* with inflammation of the gut mucosa and early recurrence of CD after ileocecal resection [56,57]. 

A trial that investigated the effects of fish oil and antioxidants (vitamins A, C, E and selenium) vs. placebo in patients with CD mainly observed decreased inflammatory markers [58]. Taking into consideration the increased risk of micronutrient deficiency (including vitamin C) [59,60], as well as the combined presence of oxidative stress and gut microbiota disturbances in the IBD population, high-dose vitamin C supplementation may be considered a highly promising nutritional intervention in this disease. Clinical trials with CD patients further investigating the microbiota-modulating role of vitamin supplements, including vitamin C, are forthcoming (www.clinicaltrials.gov, accessed on 18 June 2021; NCT04913467).

## 5. Conclusions

In conclusion, this human pilot study demonstrates that supplementation of high-dose vitamin C can manipulate the gut microbiota composition, leading to shifts of bacterial populations in the gut. This alteration of the gut microbiota composition and its associated anti-inflammatory effects with, for example, butyrate production may potentially be beneficial for a variety of diseases characterized by disturbed gut microbiota homeostasis, e.g., IBD, metabolic, cardiovascular and neurological diseases.

## Figures and Tables

**Table 1:** Baseline demographic and lifestyle characteristics of the study population (*n* = 14) consisting of healthy volunteers.

	Numbers (*n*, %) or Median [IQR]
Age (years)	22 [21;23]
Gender	
Male, *n* (%)	6 (43%)
Female, *n* (%)	8 (57%)
Length (cm)	177 [169;183]
Weight (kg)	69 [62;78]
BMI (kg/m^2^)	22.0 [21.7;23.3]
Current smoking, *n* (%)	2 (14%)
Alcohol use (units/week)	5 [3;6]
Sports activity (h/week)	3 [2;6]

Data are presented as median (interquartile range, IQR) or proportions *n* with corresponding percentages (%). Abbreviations: BMI, body mass index.

**Table 2:** Median relative abundances (%) of bacterial groups before (T0) and after (T2) two weeks of vitamin C supplementation.

Bacterial Group	T0	T2	*p*-Value
*Bacteroidetes*	5.97 [4.88;8.87]	3.09 [2.14;4.77]	**0.004**
*Ruminococci Clostridium group XIVa*	2.06 [1.28;2.82]	2.66 [1.92;3.13]	0.048
*Bifidobacterium*	1.86 [1.10;3.77]	3.29 [1.65;5.61]	0.272
*Blautia*	9.68 [7.67;11.8]	11.3 [10.1;14.5]	0.035
*Ruminococcaceae (-F. prausnitzii)*	16.0 [12.1;17.8]	13.0 [7.6;15.5]	0.035
*Enterococci*	0.40 [0.22;0.51]	0.13 [0.07;0.18]	**0.009**
*Veillonella-Dialister*	0.40 [0.04;0.96]	0.44 [0.13;1.46]	0.300
*Lachnospiraceae (other)*	12.7 [9.71;16.6]	16.2 [13.8;17.6]	**0.013**
*Rest group*	15.8 [14.2;20.0]	13.2 [10.4;16.4]	0.510
*Roseburia*	5.26 [2.77;8.81]	5.54 [4.38;9.67]	0.140
*Faecalibacterium prausnitzii*	14.9 [11.4;17.7]	12.0 [10.3;17.8]	0.551
*Gemmiger formicilis*	3.96 [2.51;6.59]	1.86 [0.81;3.68]	**0.013**
*Prevotella copri*	0.01 [0.002;3.39]	0.003 [0.001;3.01]	0.221
*Collinsella aerofaciens*	0.49 [0.26;0.98]	0.69 [0.44;1.49]	0.363
*Intestinibacter bartlettii*	0.19 [0.13;0.90]	0.14 [0.05;0.56]	0.158
*Barnesiella intestinihominis*	0.16 [0.12;0.24]	0.11 [0.06;0.16]	0.638
*Holdemanella biformis*	0.001 [0.00;0.54]	0.004 [0.00;0.85]	0.889
*Akkermansia muciniphila*	0.13 [0.02;0.28]	0.07 [0.02;0.12]	0.258
*Christensenella minuta*	0.05 [0.04;0.09]	0.03 [0.01;0.08]	0.683
*Streptococcus thermophilus*	0.51 [0.33;0.78]	0.25 [0.16;0.49]	0.096

Data are presented as median (interquartile range, IQR) of relative abundances (%) at baseline (T0) and two weeks after vitamin C supplementation (T2). Nominal *p*-value for Wilcoxon’s signed-rank test. Significances after adjustment for multiple comparisons (Benjamini–Hochberg method, FDR < 0.1) are indicated in **bold**. Abbreviations: FDR, false discovery rate; T0, baseline; T2, two weeks after vitamin C supplementation.

## Data Availability

Data is contained within the present article.
